# Light Field Angular Super-Resolution via Spatial-Angular Correlation Extracted by Deformable Convolutional Network

**DOI:** 10.3390/s25040991

**Published:** 2025-02-07

**Authors:** Daichuan Li, Rui Zhong, Yungang Yang

**Affiliations:** School of Computer Science, Central China Normal University, Wuhan 430079, China; dcli@mails.ccnu.edu.cn (D.L.); yungangyang@mails.ccnu.edu.cn (Y.Y.)

**Keywords:** light field, angular super-resolution, optical sensors, reconstruct, deep neural network

## Abstract

Light Field Angular Super-Resolution (LFASR) addresses the issue where Light Field (LF) images can not simultaneously achieve both high spatial and angular resolution due to the limited resolution of optical sensors. Since Spatial-Angular Correlation (SAC) features are closely related to the structure of LF images, its accurate and complete extraction is crucial for the quality of LF images reconstructed by the LFASR method based on Deep Neural Networks (DNNs). In low-angular resolution LF images, SAC features must be extracted from a limited number of pixels that are at a great distance from each other and exhibit strong correlations. However, existing LFASR methods based on DNNs fail to extract SAC features accurately and completely. Due to the limited receptive field, methods based on regular Convolutional Neural Networks (CNNs) are unable to capture SAC features from distant pixels, leading to incomplete SAC feature extraction. On the other hand, methods based on large convolution kernels and attention mechanisms use an excessive number of pixels to extract SAC features, resulting in insufficient accuracy in extracted SAC features. To solve this problem, we introduce Deformable Convolutional Network (DCN), which adaptively changes the position of limited sampling point using offsets, so as to extract SAC from distant pixels. In addition, in order to make the offset of DCN more accurate and further improve the accuracy of SAC features, we also propose a Multi-Maximum-Offsets Fusion DCN (MMOF-DCN). MMOF-DCN can reduce the exploration range of finding the desired offset, thereby improving the offset finding efficiency. Experiment results show that our proposed method has advantages in real-world dataset and synthetic dataset. The PSNR value in synthetic dataset which have large disparity is improved by 0.45 dB compared to existing methods.

## 1. Introduction

High-resolution Light Field (LF) image possess the unique capability to encapsulate both comprehensive spatial and angular information, making them highly valuable in applications such as Virtual Reality [1], depth estimation [2,3], and 3D reconstruction [4,5]. However, acquiring high-resolution LF image directly remains a significant challenge. Specifically, LF images are captured using microlens arrays, but the limited number of optical sensor pixels in these arrays poses a trade-off: recording light direction (angular resolution) necessitates dedicating some optical sensor pixels to capture light from different directions, leading to reduced spatial resolution. To overcome this limitation, Light Field Angular Super-Resolution (LFASR) techniques have been developed to reconstruct High Angular Resolution (HAR) LF images from Low Angular Resolution (LAR) LF images, which preserve high spatial resolution but have LAR.

**Figure 1:**
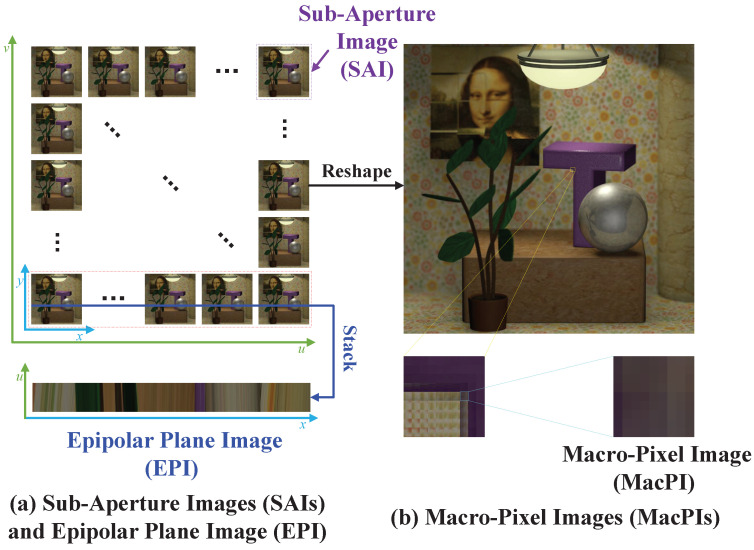
The three representations of LF image. (**a**) shows the LF image representation of Sub-Aperture Image (SAI) and Epipolar Plane Image (EPI). (**b**) shows the LF image representation of Macro-Pixel Image (MacPI). Since each pixel in an SAI comes from the same angular position, there is only a spatial relationship between the pixels within a SAI, not an angular relationship. Similarly, since the pixels in each MacPI come from the same spatial position, there is only an angular relationship between the pixels within a MacPI, not a spatial relationship. The Epipolar Plane Image (EPI) is generated by extracting and stacking SAIs along designated spatial and angular dimensions. This imaging modality inherently encapsulates both spatial and angular information, thereby exhibiting spatial-angular correlation.

In recent years, Deep Neural Networks (DNNs) have shown strong reconstructive ability in LFASR [6,7,8,9,10,11,12]. Accurate and complete extraction of the main features in LAR LF images is crucial for improving the quality of HAR LF images reconstructed using DNN-based LFASR methods. As shown in Figure 1, these main features typically include spatial features extracted from Sub-Aperture Image (SAI), angular features extracted from Macro-Pixel Image (MacPI), and Epipolar Plane Image (EPI) features extracted from EPI.

**Figure 2:**
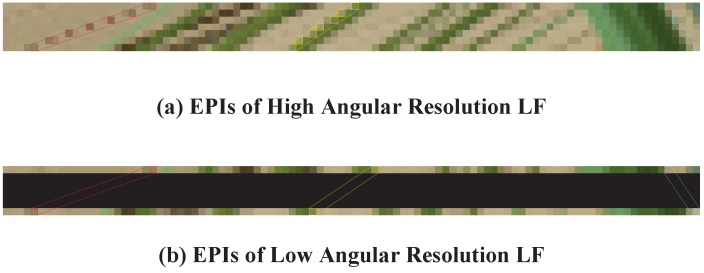
The EPIs of HAR LF image and LAR LF image. (**a**) shows the EPIs of the HAR LF images. In (**b**), we simulate the EPIs of the LAR LF image by obscuring the views in the EPIs of HAR LF image with black areas. We use three different colored slashes in the EPIs to represent three different SACs. Arranged in descending order of length, their corresponding colors are red, yellow, and blue, respectively. The pixels within each rectangle in the SAC can be used to extract SAC features. As observed, extracting SAC features from LAR LF images necessitates the involvement of more distant pixels. Additionally, the distance between the pixels required to extract different SAC feature varies.

Although the existing DNN-based LFASR methods are highly effective, they overlook the fact that EPI contains Spatial-Angular Correlation (SAC) in LF images and fail to accurately and completely extract SAC features. This results in artifacts and blurring in the reconstructed HAR LF images. SAC features are closely related to the disparity in LF images [10], and their accurate and complete extraction can significantly improve the quality of reconstructed HAR LF images. As shown in Figure 2, in the EPIs of LAR LF images, SAC feature must be extracted from a limited number of strongly correlated pixels at greater distances due to the loss of angular resolution. Due to the limited receptive field, existing methods based on regular Convolutional Neural Networks (CNNs) [10] cannot completely extract SAC features from pixels at greater distances. Methods using large convolution kernels [12] and attention mechanisms [9] fail to accurately extract SAC features, as they establish relationships across a large number of pixels and introduce many weakly correlated pixels for feature extraction. Therefore, it is essential to design an LFASR method capable of extracting SAC features using a limited number of highly correlated pixels at greater distances.

To solve this problem, we introduce Deformable Convolutional Network (DCN) [13] on the EPI to extract SAC features, enabling adaptive adjustment of sampling point positions through learnable offsets. Unlike regular CNNs constrained by small fixed convolutional kernels, DCNs dynamically adapt their receptive fields based on the distances between pixels needed for SAC feature extraction. Additionally, unlike methods that use large convolution kernels or attention mechanisms, DCNs focus only on a limited number of strongly correlated pixels, effectively preventing SAC feature extraction from being compromised by the inclusion of weakly correlated pixels.

Although DCN is capable of efficiently selecting a limited number of the most relevant points over a large range to extract SAC features, its fixed maximum offset imposes limitations, leading to inaccuracies and incomplete SAC feature extraction when SAC features that involve varying pixel distances. As shown in Figure 2b, the distances between pixels required for SAC feature extraction in LF scenes vary due to the non-uniform disparities of objects in the scene. This characteristic leads to the following issues when directly using a DCN with a fixed maximum offset: if a DCN with a larger maximum offset is used to extract SAC features with shorter distances, the accuracy of SAC extraction decreases; conversely, if a DCN with a smaller maximum offset is used to extract SAC features with great distances, the SAC cannot be extracted.

To solve this problem, we design a Multi-Maximum-Offsets Fusion DCN (MMOF-DCN). In this design, we first analyzed the distribution of sampling point offsets needed to extract different SAC features from LF images. Based on this analysis, we created multiple DCNs with different maximum offsets, which are then fused using an Adaptive Weight Mechanism (AWM). This design allows the DCN to more accurately focus on the appropriate offset range for each SAC feature, improving the precision of the extracted offsets and, ultimately, enhancing the accuracy of SAC feature extraction.

In summary, the contributions of this paper are as follows:

- To address the difficulty in extracting SAC features on the EPI of LAR LF images due to the increased distance between pixels required for SAC extraction, we introduce DCN to the EPI to adaptively adjust the sampling point positions according to the distance between the required pixels for SAC extraction.
- To address the issue that DCN with a fixed maximum offset cannot accurately and completely extract SAC features with varying pixel distances in LF scenes, we employ the Canny operator to compute and analyze the offsets required by DCN for extracting different SAC features. Based on this analysis, we propose a MMOF-DCN method. This method adaptively selects more precise sampling point offsets for different SAC features, thereby significantly enhancing the accuracy of SAC feature extraction.
- We conducted extensive experiments on both real-world and synthetic datasets. Experimental results show that our method outperforms existing methods under both quantitative and qualitative conditions. The PSNR on synthetic dataset with large disparity is 0.45 dB higher than that of existing methods.

## 2. Related Work

LF angular super-resolution methods based on DNNs are usually divided into depend disparity estimation methods and non-depend disparity estimation methods. The depend disparity estimation methods [14,15,16,17,18] usually use deep learning techniques to estimate the disparity of LF images and combine it with warp operations to reconstruct novel views. Kalantari et al. [14] used CNN to implement disparity estimation and color estimation. Jin et al. [15] predicted a disparity map for each view and introduced a blending strategy to recover the geometry structure of the EPIs. Ko et al. [16] extracted multi-view features by disparity estimation and refined the features using an adaptive blending method. In LF scenes without occlusion, disparity estimation is generally accurate, allowing the depend disparity estimation methods to effectively reconstruct novel views. However, when occlusion occurs in LF scenes, the accuracy of disparity estimation significantly decreases. This results in degraded image quality of the reconstructed HAR LF images.

The non-depend disparity estimation methods [6,7,8,9,10,11,12,19,20,21,22,23,24] usually use deep learning techniques to extract features from each feature domain of LF images and combine these features with upsampling methods to reconstruct HAR LF images. Yeung et al. [7] alternately use spatial convolution and angular convolution to simulate 4D convolution to extract features from LF images. Wang et al. [19] proposed a pseudo 4DCNN to achieve LFASR task in horizontal and vertical directions step by step. Wu et al. [8] proposed an anti-aliasing framework based on Laplacian pyramid to optimize the output ASR LF image. Wang et al. [10] proposed a decoupling mechanism for LF images. They proposed different convolutions on MacPIs to extract spatial features, angular features and EPI features. Liu et al. [11] used CNN to achieve ASR, and then used angular Transformer and deblurring network to optimize the blurred ASR LF image. Xia et al. [12] replace global modeling in Transformer with large convolutional kernels to build local but broader relationships that represent non-local spatial features, angular features, and EPI features. However, these methods fail to recognize that the features on the EPI are SAC features associated with disparity. Additionally, the distance between the pixels required to extract SAC features becomes distant in LAR LF images, and is only related to a limited number of points. Without accurate extraction of SAC features, it is impossible to preserve a consistent disparity structure in the reconstructed HAR LF image, resulting in noticeable artifacts and blurring.

## 3. SAC Feature Analysis

### 3.1. The Definition and Significance of the SAC Feature

For any point (a given object point) $p_{i}$ in 3D scene, its imaging point on the sensor is recorded as $\left(x_{i},y_{i},v_{i}\right)$ triplet, where $\left(x_{i},y_{i}\right)$ is the imaging position coordinate and $v_{i}$ is the pixel value. At *n* different views, disparity means that $p_{i}$ is projected or imaged to different positions on *n* SAIs. At the same time, the pixel values at these *n* different imaging positions are correlated, called SAC.

SAC is closely related to disparity. The cause of disparity is related to the imaging principle of a camera which projects a 3D object from the world coordinate system onto the camera’s focal plane. This process essentially involves coordinate transformations through a series of transformation matrix multiplications, ultimately yielding the pixel coordinates in the image. Since the transformation matrices change with the camera position, different views will result in different pixel coordinates [25,26]. When creating a pair of images from two adjacent cameras locations on a certain plane, a given object point $p_{i}$ will project to different pixel locations in the two images, potentially several pixels apart. The distance between these two projected locations is related to disparity. Moreover, the pixel values at these two locations are correlated. In LF images, this correlation of pixel values aligns with SAC.

Using the EPI to study SAC features is highly beneficial, as the projections of an object from different views in the EPI form a distinct slash line. As shown in Figure 1a, the EPI is typically created by cutting the LF image along fixed spatial and angular directions according to the view sequence and then stacking the slices. In the EPI, an object is projected onto views in a fixed direction. Due to the consistent disparity of the object, the projection coordinates change in a regular pattern. When these projections are stacked in view order, as shown in Figure 2, the EPI of LF image reveals that pixels sharing the same SAC align along a obvious slash line. The slope of this slash line directly corresponds to the disparity of objects in the LF scene [10]. Since the disparity of objects within an LF scene remains constant, the SAC of the same object is invariant across both HAR LF images and LAR LF images. Consequently, accurately extracting SAC features from LAR LF images is critical for ensuring that the reconstructed HAR LF images maintain a complete and consistent disparity structure.

### 3.2. Limitations of Existing Methods in SAC Feature Extraction

As shown in Figure 2a, in the EPI of HAR LF images, the imaging coordinates of pixels with the same SAC across adjacent views tend to be closely aligned. This proximity can be effectively utilized for extracting SAC features. However, as shown in Figure 2b, reducing angular resolution causes adjacent views in the EPI of an LAR LF image to correspond to views that are distant apart in the HAR LF image. In this case, the sampling point positions need to be extended to extract the complete SAC features.

Existing methods do not account for the unique characteristics of SAC features in LAR LF images, which often results in incomplete SAC feature extraction. When the distance between the pixels required for extracting SAC features increases, methods based on regular CNNs [8,10] are limited by their small convolutional kernels, making it difficult to accurately extract SAC features from distant pixels. To address this, some researchers have used large convolution kernels [12] and attention mechanisms [9] to establish connections among a large set of pixels—or even all pixels—within the EPI, including those pixels that are distant. However, establishing such extensive pixel connections presents two main challenges. First, it significantly increases computational complexity and memory requirements. Second, for any object in LF scenes, typically only a single pixel is projected in each view. While adjacent pixels within the same view may exhibit spatial correlation due to the sufficient spatial resolution of LAR LF images, most other pixels show weak correlations with this pixel. Including these weakly correlated pixels can reduce the accuracy of SAC feature extraction.

Therefore, we propose using DCN to extract SAC features. This approach can extract SAC features from distant pixels while ensuring that these pixels are the most relevant for the task.

## 4. Method

### 4.1. Overview

**Figure 3:**
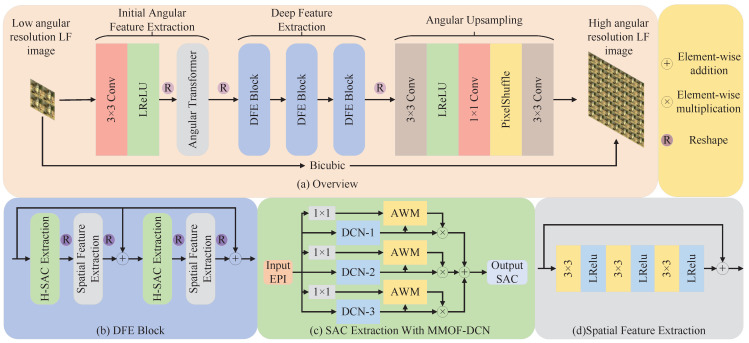
Our proposed network architecture. (**a**) shows the overview of our proposed network. (**b**) displays the detail of the Deep Feature Extraction (DFE) block. (**c**) demonstrates the basic framework of the Spatial-Angular Correlation (SAC) Extraction with Multi-Maximum-Offsets Fusion Deformable Convolutional Network (MMOF-DCN). Furthermore, AWM means Adaptive Weight Mechanism. (**d**) shows the detail of the Spatial Feature Extraction (SFE).

In network design, we focus primarily on the accurate SAC feature extraction. However, inspired by previous studies [10,12,23] that use LF essential features, we also introduce angular feature extraction and spatial feature extraction. As shown in Figure 3, for the LFASR task of a×a→A×A, we use an LAR LF image $L_{LAR}\in R^{a\times a\times H\times W}$ with an angular resolution of *a* as the input and reconstruct an HAR LF image $L_{HAR}\in R^{A\times A\times H\times W}$ with an angular resolution of *A*. The proposed network consists of three main components: Initial Angular Feature Extraction (IAFE), Deep Feature Extraction (DFE), and Angular Upsampling.

Firstly, the $L_{\mathrm{LAR}}$ is input into IAFE, which consists of an initial convolution and an angular Transformer. In the IAFE, $L_{\mathrm{LAR}}$ realizes channel expansion through the initial convolution and then use the angular Transformer to capture underlying angular information, thereby obtaining the initial feature map $F_{\mathrm{init}}\in R^{a\times a\times C\times H\times W}$. Secondly, $F_{\mathrm{init}}$ is passed through DFE, which contains three DFE Blocks. As shown in Figure 3b, each DFE Block includes a SAC Feature Extraction (SACFE) unit—built with DCNs fused with multiple maximum offsets and a Spatial Feature Extraction (SFE) unit, comprising convolutional layers with residual connections. This design extracts SAC features and spatial features from the LAR LF image. After passing through the DFE, we obtain the deep LF feature map $F_{\mathrm{DFE}}\in R^{a\times a\times C\times H\times W}$. Finally, $F_{\mathrm{DFE}}$ is input into Angular Upsampling. Angular Upsampling combines multiple convolutions and PixelShuffle to establish a connection between $F_{\mathrm{DFE}}$ and the HAR LF image, then reconstructing the HAR LF image $L_{\mathrm{HAR}}\in R^{A\times A\times H\times W}$. 

### 4.2. Initial Angular Feature Extraction

IAFE takes $L_{\mathrm{LAR}}$ as input and outputs the initial feature map $F_{\mathrm{init}}$. As shown in Figure 3b, $L_{\mathrm{LAR}}$ is processed by the initial convolution, which includes a convolution with a kernel size of 1×1 and a LeakyReLU activation. This operation expands the channel dimensions of input to *C*, producing $L_{\mathrm{init}}\in R^{a\times a\times C\times H\times W}$. Next, $L_{\mathrm{init}}$ is reshaped into the MacPI, enabling more efficient operations in the angular domain. The reshaped $L_{\mathrm{init}}$ is then passed into the Angular Feature Extraction (AFE) module. Here, we use the angular Transformer proposed by Cong et al. [27], which effectively utilizes dynamic view position encoding to capture global angular information. Finally, the angular feature map produced by the AFE is reshaped back into the SAI, resulting in the initial feature map $F_{\mathrm{init}}$ enriched with angular features.

### 4.3. Deep Feature Extraction

DFE takes $F_{\mathrm{init}}$ as input and outputs the LF deep feature map $F_{\mathrm{DFE}}$. As shown in Figure 3a, the DFE is composed of three cascaded DFE Blocks. Each DFE block consists of SFE and SACFE. The SFE, as shown in Figure 3d, consists of three convolutions with kernels size of 3×3, each followed by an LReLU activation. Each convolution is activated by an LReLU layer and outputs via a residual connection. As shown in Figure 3c, in SACFE, we employed our proposed MMOF-DCN to extract SAC features. Given that the EPI has both horizontal and vertical directions with similar characteristics, we adopt a similar structure for extracting SAC features in both directions within each DFE Block. Specifically, for each DFE Block, the input feature map alternates between SACFE and SFE to extract SAC features and spatial features, which are then combined with the input via residual connections. After passing through three DFE Blocks, DFE extracts spatial and SAC features from $F_{\mathrm{init}}$ and integrates them with the angular features contained in $F_{\mathrm{init}}$ to form the LF deep feature map $F_{\mathrm{DFE}}$.

### 4.4. SAC Feature Extraction

#### 4.4.1. DCN for SAC Feature Extraction

**Figure 4:**
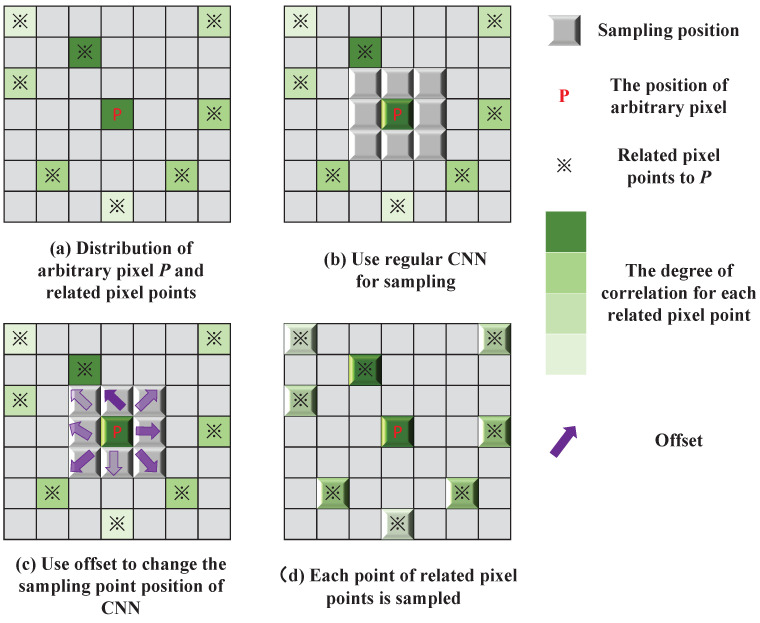
The process by which DCN adaptively changes the sampling point positions of the CNN using offset. (**a**–**d**) show the sampling point positions from unable to cover related pixel points to covering all related pixel points step by step.

In order to extract SAC features using the limited pixel points on EPI that are most relevant to SAC, inspired by Wang et al. [13], we introduced DCN. As shown in Figure 4, DCN differs from regular convolution by adaptively adjusting the sampling point positions using offsets, which allows for a flexible change in the receptive field of convolution and extended sampling point distance. With this design, DCN can not only extract SAC features from pixels at greater distances but also utilize only the most strongly correlated pixels, avoiding issues introduced by irrelevant points.

Since horizontal SAC features and vertical SAC features have similar properties, differing only in direction, we describe the process of DCN extracting SAC features in the horizontal direction as a general case. As SAC features are extracted from the EPI, the input is first reshaped into horizontal EPI, denoted as $F_{\mathrm{HEPI}}\in R^{(W\times v)\times C\times H\times u}$, to facilitate the extraction of horizontal SAC feature. For any pixel at coordinate p(i,j) in $F_{\mathrm{HEPI}}$, SAC features are extracted via DCN can be formulated as:

(1) $$F_{H-SAC}\left(i,j\right)=\sum_{t=1}^{T}\sum_{k=1}^{K}w_{t}m_{tk}F_{HEPI}\left(p+p_{k}+\Delta p_{tk}\right),i\in H,j\in u,$$

where $F_{H-SAC}\left(i,j\right)$ denotes SAC features extracted at the coordinate (i,j). *T* represents the total number of groups into which the features are divided along the channel dimension. This helps improve the efficiency of feature extraction [28]. *K* represents the total number of sampling points. $w_{t}$ and $m_{tk}$ respectively represent the channel attention weight and the sampling point positions attention weight. This mechanism adaptively assigns weights to channels and the importance of each sampling points, optimizing the utilization of information from the sampled points. The set $p_{k}=\left\{\left(-1,-1\right),\left(-1,0\right),\cdots ,\left(-1,-1\right)\right\},k\in K$ represents the initial position of each sampling point. $\Delta p_{tk}$ represents the offset for the *k*-th sampling point in channel *t*.

#### 4.4.2. Multi-Maximum-Offsets Fusion DCN

In order to enable DCN to extract more accurate SAC features of various objects in LF scenes, we introduce the MMOF-DCN. The aim is to enhance the precision of the offsets by reducing the search space for the necessary offsets in the DCN when handling different SAC, thereby improving the overall accuracy of the SAC features. We have designed MMOF-DCN through three steps: offset distribution pattern statistics, setting multiple-maximum-offsets DCN using the distribution patterns, and DCN adaptive weight fusion.

**Figure 5:**
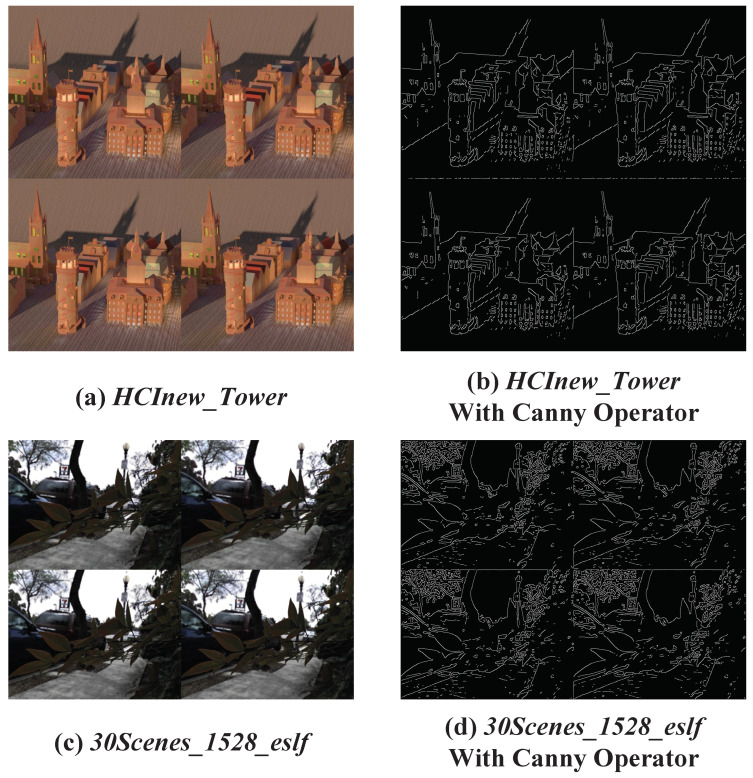
The process of edge detection of LAR LF image by Canny operator. (**a**,**b**) shows the edge detection process in the Synthetic dataset, and (**c**,**d**) shows the edge detection process in the real world dataset.

**Figure 6:**
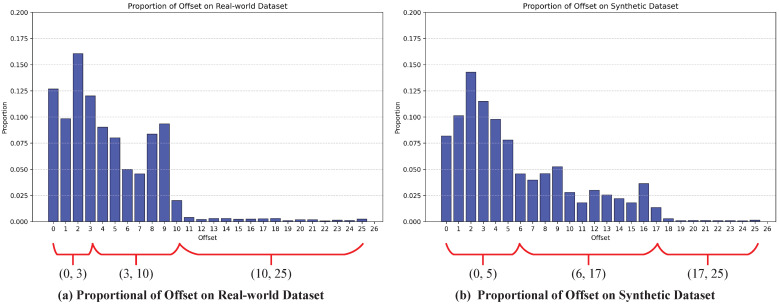
The proportional of offset required for accurate extraction of SAC of LARLF images randomly sampled by training sets of Real-world dataset and synthetic dataset data sets. We have marked three main divisions in the diagram.

The first step is offset distribution pattern statistics. We describe this process using the 2×2→7×7 LFASR task. Based on previous work [10,12,23], we selected real-world dataset (including 30 Scenes [14], Occlusion [29], and Reflective [29]) and synthetic dataset (including HCInew [30] and HCIold [31]) for the study. Specifically, we randomly selected 20 HAR LF images from the training set of the real-world dataset and 5 from the synthetic dataset, and constructed LAR LF images by removing several adjacent views. In LF scenes, the disparity of points on the same object is generally similar, and since our goal is to analyze the distribution range of the maximum offsets, precise calculation of the offsets for all points is unnecessary. As shown in Figure 5, we applied the Canny operator to perform edge detection on each randomly selected LAR LF image, which helped separate the different objects in the LF scene. We then extracted the horizontal EPI and vertical EPI from these edge-detected LF scenes. For any edge point in a specific view of the EPI, we identified the closest matching edge point in the neighboring views based on pixel value. Finally, as shown in Figure 6a,b, we performed a statistical analysis of the offsets required for the LF images in both datasets.

The second step is setting multiple-maximum-offsets DCN using the distribution patterns. As shown in Figure 6a,b, in both datasets, the offsets required to capture pixels with identical SAC features generally fall into three intervals: the most common offset range $L_{1}\;\left(0,l_{1}\right)$, the moderately common offset range $L_{2}\;\left(l_{1},l_{2}\right)$, and the least common offset range $L_{3}\;\left(l_{2},l_{3}\right)$. For the 2×2→7×7 LFASR task, in the real-world dataset, $l_{1}=3,\;l_{2}=10,$ and $l_{3}=25$; while in the synthetic dataset, $l_{1}=5,\;l_{2}=17,$ and $l_{3}=25$. Based on these analyses, we configure three DCNs—$D_{1},\;D_{2}$, and $D_{3}$—each with a maximum offset of $l_{1},\;l_{2}$, and $l_{3}$, respectively, to extract SAC features using different maximum offsets.

**Figure 7:**
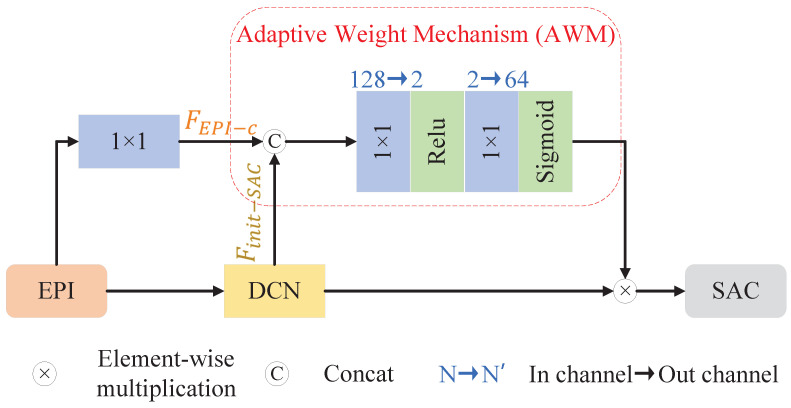
Details of the *i* branch of MMOF-DCN. In the figure, we assume that the number of input channels for EPI and output channels for DCN is 64.

The third step is DCN adaptive weight fusion. In this phase, SAC features are first extracted using DCNs with different maximum offsets, and then weighted through an AWM before being fused. Specifically, for the feature maps $F_{EPI}$, we first use $D_{1}$, $D_{2}$, and $D_{3}$ to extract the initial SAC features, resulting in $F_{init-SAC}^{i}$ for i∈1,2,3. Secondly, we applying a convolution with a kernel size of 1×1 to $F_{EPI}$ for channel-wise interaction, producing $F_{EPI-c}^{i}$ for i∈1,2,3. It is important to note that the convolution weights used in this interaction are shared, which ensures consistency in the subsequent steps when utilizing $F_{EPI-c}$. Thirdly, $F_{EPI-c}^{i}$ and $F_{init-SAC}^{i}$ are input into the *i*-th AWM. As shown in Figure 7, in the AWM, $F_{EPI-c}^{i}$ and $F_{init-SAC}^{i}$ are concatenated along the channel dimension, and a learnable weight mechanism is employed to adaptively adjust the accuracy of $F_{init-SAC}^{i}$. The AWM consists of two convolutions with kernels size of 1×1, a ReLU activation layer, and a sigmoid function. The first convolution maps from 2C channels to 2 channels, and the second maps from 2 to *C* channels. The two convolutions allow for determining the SAC feature weights based solely on the pixel information, without interference from other pixels in the EPI. After obtaining the weights from the AWM, we multiply them with $F_{init-SAC}^{i}$ to yield the weighted SAC features $F_{w-SAC}^{i}$ for i∈1,2,3. Finally, all $F_{w-SAC}^{i}$ are summed, and a convolution with a kernel size of 1×1 and *C* output channels is applied for channel-wise interaction, producing the precise SAC feature map $F_{SAC}$:

(2) $$F_{SAC}=H_{1\times 1}\left(\sum_{i}F_{init}^{i}*AWM\left(F_{init-SAC}^{i},F_{EPI-c}\right)\right),i\in 1,2,3.$$

### 4.5. Angular Upsampling

After extracting the deep features $F_{\mathrm{DDF}}$ from the LAR LF image, we reconstruct the HAR LF image using a combination of convolutional operations and PixelShuffle, following previously methods [10,12,23]. Specifically, $F_{\mathrm{DDF}}$ is first reshaped into the MacPI format. Secondly, it is processed by a full-angular convolution with a kernel size of a×a to integrate features in the angular domain. Thirdly, a convolution with a kernel size of 1×1 is applied to expand the channel dimensions to A×A×C, followed by PixelShuffle for redistribution. Fourthly, a spatial convolution with a kernel size of 3×3 maps the channels back to their original dimension, generating an initial high angular resolution LF image $L_{init-HAR}$. Finally, bicubic interpolation is applied to $L_{LAR}$ for blurry angular super-resolution, and the result is added to $L_{init-HAR}$ to produce the final HAR LF image:

(3) $$L_{HAR}=L_{init-HAR}+Bicubic\left(L_{LAR}\right).$$

## 5. Experiment

To evaluate the performance of our proposed method across diverse LF scenes, we followed previous works [10,12,15] and conducted training and testing on both real-world dataset and synthetic dataset. We have compared our proposed method with existing methods. In addition, we also conducted a number of ablation studies to verify the effectiveness of the various components of our proposed method.

### 5.1. Setup

#### 5.1.1. Datasets

In real-world dataset, three public datasets are used, which are 30Scenes [14], Occlusion [29] and Reflective [29]. We use 100 LF images from 30Scenes for training and 70 LF images for testing. These test LF images include 30 LF images from 30Scenes, 25 LF images from Occlusion, and 15 LF images from Reflective.

In the synthetic dataset, two public datasets are used, which are HCInew [30] and HCIold [31]. We use 20 LF images from HCInew for training, and 4 LF images from HCInew and 5 LF images from HCIold for testing. It is worth noting that the distance between the cameras used to obtain HCInew and HCIold is relatively large, so the distance between each view in the obtained LF image is large. Therefore, compared to the real-world dataset, synthetic dataset has larger disparity and is more challenging.

#### 5.1.2. Implement Details

We implement our method in PyTorch. Our method is trained and tested on a single NVIDIA 3090 GPU. For the channel number *C* of our proposed network, except for the version *C* is equal to 32 introduced in Section 5.2.3, the conventional version *C* is equal to 32 was used in all the other experiments.

During training, the training images are cropped to 64×64 size and the batch size is set to 1. Since L1 Loss is more robust to outliers [32], we use it as the loss function for training. The initial learning rate is set to 1e-4, decayed every 25 epochs, and the decay rate is 0.5. Similar to previous works [9,10,12,14,15], we represent LF images with YCBCR and evaluate the quality of LF angular super-resolution on the Y channel. For the CBCR channel, we apply Bicubic interpolation to upsample them.

### 5.2. Comparison with Existing Methods

We choose six representative existing methods as the comparison methods. They are Kalantari et al. [14], Yeung et al. [7], Jin et al. [15], SAANet [9], DistgASR [10], and Xia et al. [12]. We use 2×2→7×7 LFASR task and 3×3→9×9 LFASR task to evaluate the superiority of our proposed approach. To compare the difficulty of the LFASR tasks, we can compare the upsampling scale λ of the angular dimension in the LFASR tasks. Specifically, for the a×a→A×A LFASR task, the upsampling scale λ is defined as $\lambda =\frac{A-1}{a-1}$ [9]. A larger value of λ means that each view in the LAR LF image must reconstruct more views from the HAR LF image, which increases the complexity of the LFASR task. This means that the 2×2→7×7 LFASR task (6× Upsampling) is more challenging to reconstruction than the 3×3→9×9 LFASR task (4× Upsampling). Therefore, we validate the superiority of our method for challenging large disparity LFASR tasks with the former, and the generality of our method for routine LFASR tasks with the latter. For each task, we evaluated it separately using quantitative analysis and qualitative analysis. In quantitative analysis, PSNR and SSIM are used as evaluation criteria. In qualitative analysis, we conducted three presentations to evaluate, error map, two details and EPI reconstruction, respectively. In addition, we also compare the number of parameters to evaluate the efficiency of parameter use of our proposed method.

#### 5.2.1. 2×2→7×7 LFASR Task

The comparative experiment commences with a systematic quantitative analysis. As shown in Table 1 and Table 2, our proposed method achieves higher PSNR and SSIM on both real-world and synthetic datasets compared to existing methods. Because Yeung et al. [7] only used spatial and angular features without SAC features, they could not use all essential LF features to reconstruct HAR LF images. In occluded LF scenes, methods that depend on disparity estimation, such as those by Kalantari et al. [14] and Jin et al. [15], exhibit noticeably lower reconstruction quality compared to our approach. This is due to the reduced accuracy of disparity estimation caused by occlusions, which significantly degrades the reconstruction results.

On synthetic dataset with large disparity, our proposed method achieves an average PSNR that is 0.45 dB higher than existing method. SAANet rely on global attention mechanisms for SAC feature extraction leads to the inclusion of numerous irrelevant points, thereby reducing the precision of SAC features. Additionally, their method overlooks other essential LF image features. These have led to poor reconstruction quality of their methods. While DistgASR and Xia et al. [12] incorporate essential LF features, their methods fail to effectively extract SAC features. DistgASR is limited by regular CNN, which captures overly localized features on EPIs and struggles with accurate SAC feature extraction in large-disparity LF scenes. Xia et al. [12] use convolutions with large kernel introduces excessive irrelevant points, causing significant errors. In contrast, our proposed method not only expands the receptive field for SAC feature extraction but also selectively focuses on a limited set of the most relevant points. This enables accurate SAC feature extraction even in LF scenes with large disparity, leading to superior reconstruction performance.

**Figure 8:**
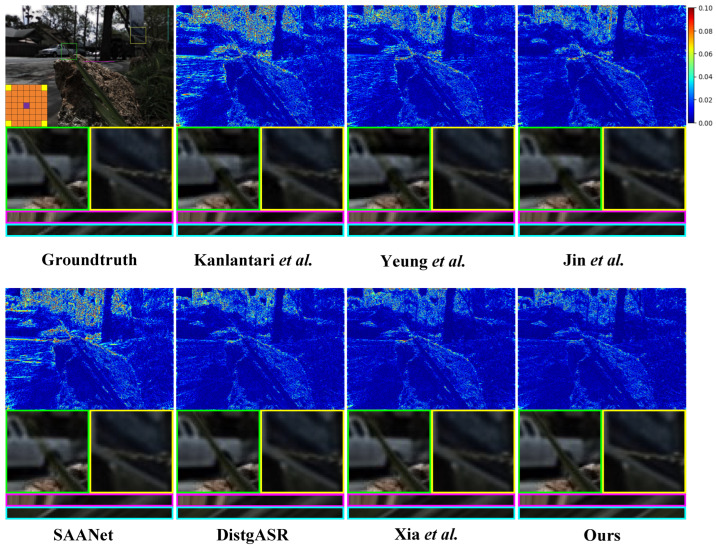
Visual comparison of the of 2×2→7×7 LFASR task in real-world LF scene. We choose the 30scenes_Rocks [14] for comparison and show it with the central view of (4,4). The comparison shows the error map, two local details, a H-EPI and a V-EPI ([7,9,10,12,14,15]).

**Figure 9:**
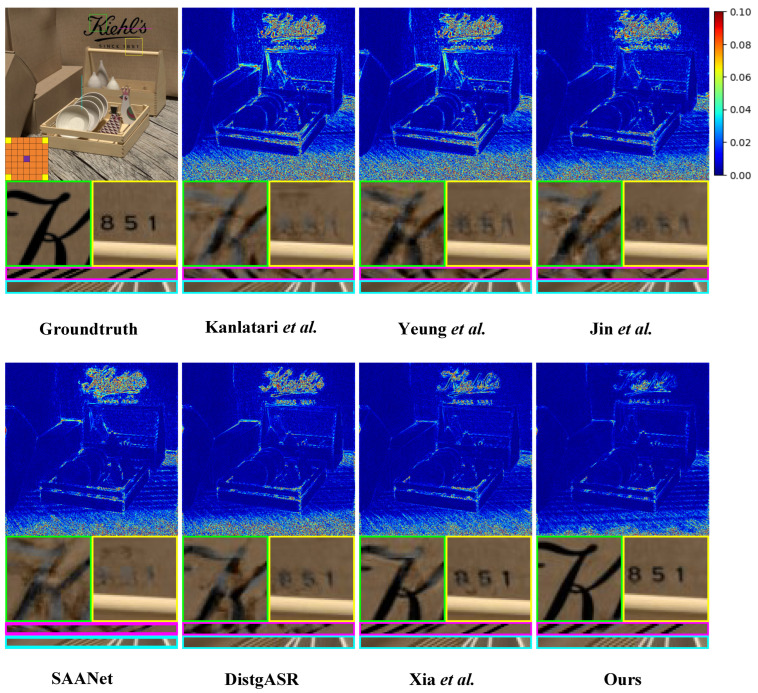
Visual comparison of the of 2×2→7×7 LFASR task in synthetic LF scene. We select the HCInew_dishes [30] for comparison and show it with the central view of (4,4). The comparison shows the error map, two local details, a H-EPI and a V-EPI ([7,9,10,12,14,15]).

**Figure 10:**
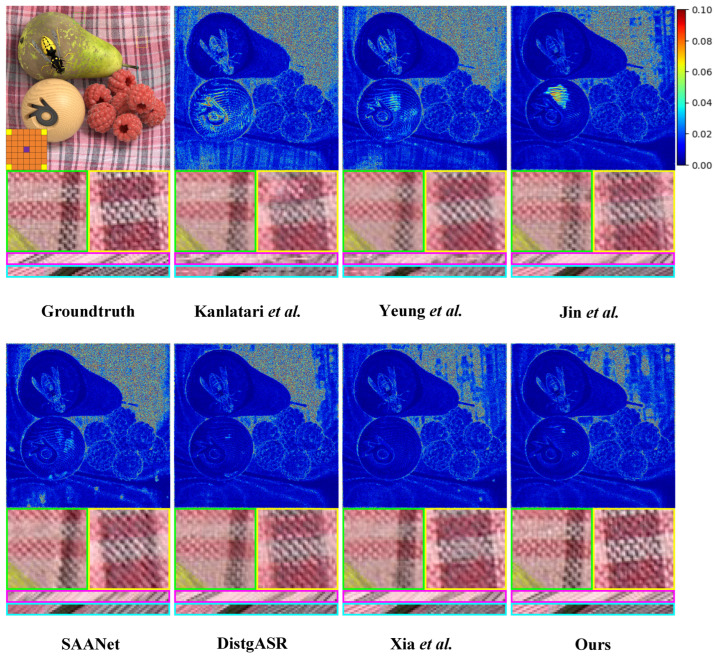
Visual comparison of the of 2×2→7×7 LFASR task in synthetic LF scene. We select the HCIold_stillLifes [31] for comparison and show it with the central view of (4,4). The comparison shows the error map, two local details, a H-EPI and a V-EPI ([7,9,10,12,14,15]).

Furthermore, we augment our comparative experiment through comprehensive qualitative analysis. As shown in Figure 8, Figure 9 and Figure 10, we illustrate the visual comparisons between our proposed method and other methods on both real-world and synthetic LF scenes. Since synthetic LF scenes are more challenging, we selected one image each from the HCInew and HCIold datasets for analysis. In real-world LF scenes, methods that depend on disparity estimation, such as Kalantari et al. [14] and Jin et al. [15], struggle to determine whether the rear vehicle is occluded by grass. Compared to Yeung et al. [7], our method produces significantly more accurate error maps, indicating clearer reconstructed images.

In synthetic LF scenes, DistgASR relies on regular CNNs to extract SAC features based on adjacent pixels, which overemphasizes local neighborhood information. This leads to noticeable artifacts and blurring in areas containing letters and numbers in the images reconstructed from HCInew. For EPI reconstruction, both Xia et al. [12] using convolutions with large kernel, and SAANet employing global attention, introduce excessive pixel points. Consequently, the disparity structure is poorly preserved in the EPIs reconstructed from HCInew and HCIold, leading to extensive blurry regions. In contrast, our method effectively preserves the EPI structure by utilizing only a limited number of the most relevant pixels for SAC features.

#### 5.2.2. 3×3→9×9 LFASR Task

In the 3×3→9×9 LFASR task, we only choose SAANet, DistgASR, and Xia et al. [12] as the comparison methods for two primary reasons. First, we previously analyzed the limitations of Kalantari et al. [14], Yeung et al. [7], and Jin et al. [15] during the 2×2→7×7 LFASR task. These limitations, which arise from factors such as LF scene composition (e.g., occlusion) and insufficient feature utilization, are not affected by the degree of angular resolution reduction. Second, SAANet, DistgASR, and Xia et al. [12] each represent different strategies for extracting SAC features from EPIs—global attention mechanisms, regular CNNs, and convolutions with large kernel. Given the characteristics of SAC features in LAR LF images, as discussed earlier, their feature extraction capabilities are influenced by the reduction of angular resolution.

We also employ quantitative analysis to conduct our comparative experiments. As shown in Table 3 and Table 4, our method achieves higher PSNR and SSIM on both real-world and synthetic datasets compared to existing methods. In the 3×3→9×9 LFASR task, the limited loss of views results in shorter distances between pixels belonging to the same SAC on the EPI. This makes CNNs-based SAC extraction methods like DistgASR more likely to accurately capture SAC features, leading to a substantial performance improvement compared to the 2×2→7×7 LFASR task. However, DistgASR still struggles with capturing points exhibiting larger disparities, which limits its reconstruction quality compared to our method that effectively explores a broader receptive field. While SAANet and Xia et al. [12] also benefit from the increased number of views, allowing them to capture more pixels belonging to the same SAC and thereby improving reconstruction performance, these methods also incorporate more irrelevant pixels. As a result, their reconstruction quality falls short of our method, which focuses solely on the most relevant pixels for SAC feature extraction.

**Figure 11:**
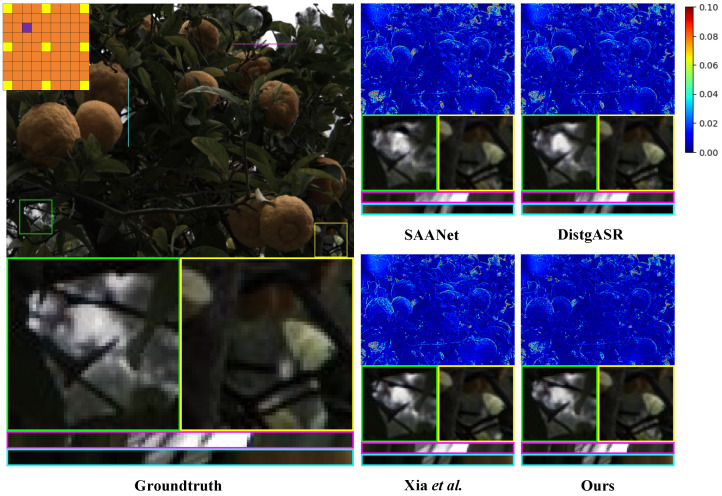
Visual comparison of the of 3×3→9×9 LFASR task in real-world LF scene. We select the Occlusion_44_eslf [29] for comparison and show it with the central view of (3,3). The comparison shows the error map, two local details, a H-EPI and a V-EPI ([9,10,12]).

**Figure 12:**
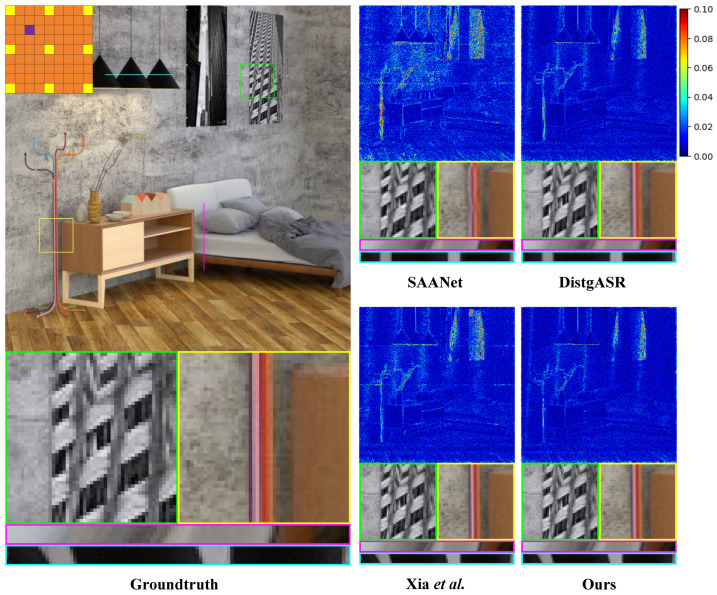
Visual comparison of the of 3×3→9×9 LFASR task in synthetic LF scene. We select the HCInew_bedroom [30] for comparison and show it with the central view of (3,3). The comparison shows the error map, two local details, a H-EPI and a V-EPI ([9,10,12]).

The qualitative analysis substantiates the superiority of our proposed method over existing methods. As shown in Figure 11 and Figure 12, we illustrate the visual comparisons between our method and other methods on both real-world and synthetic LF scenes. In the detailed display of the real-world LF scene, the reconstruction by DistgASR shows noticeable blurring, particularly at the junction of the branches and the background on the left, as well as in adjacent regions. In the synthetic LF scene, the EPI reconstruction by SAANet and Xia et al. [12] exhibits significant directional errors. This problem stems from the SAC features they extracted being influenced by an excessive number of irrelevant points, which hampers the accurate extraction of SAC features.

#### 5.2.3. Parameter Number Comparison

To better analysis the use of parameters and make a fair comparison with other methods, we evaluate the effectiveness of our proposed method using two versions with different parameter sizes: C=32 and C=64. As shown in Table 5, we present the parameter size and average evaluation results for the synthetic dataset in the 2×2→7×7 LFASR task. Compared to the methods with smaller parameter sizes such as Yeung et al. [7], Jin et al. [15], and SAANet, our C=32 version not only has a similar parameter size but also achieves a 1.02 dB increase in PSNR. When compared to methods with moderate parameter sizes, such as DistgASR, our C=32 version uses fewer parameters while still achieving a 0.3 dB improvement in PSNR. In the comparison with larger models, our C=64 version, with similar parameters count to Xia et al. [12], achieves a 0.45 dB increase in PSNR. These parameter comparisons highlight the efficient utilization of parameters in our proposed method.

### 5.3. Ablation Study

In this section, we verify the validity of the various components of our proposed method through different ablation studies. We ensured the consistency of the number of parameters by adjusting the number of channels. In addition, our ablation studies were performed on challenging 2×2→7×7 LFASR task, as well as on synthetic dataset with large disparity.

#### 5.3.1. Effectiveness of MMOF-DCN in SAC Feature Extraction Compared with Other Models

To verify the effectiveness of MMOF-DCN in accurately extracting SAC feature, we introduced three representative models that are commonly used on EPI and proposed three variants based on these models, which are named SAC-R-CNN, SAC-Transformer, and SAC-B-CNN. Specifically, each variant substitutes the MMOF-DCN with a different model: in SAC-R-CNN, we replace it with the Regular CNN used in DistgASR [10] to capture EPI features on MacPI; SAC-Transformer substitutes the EPI Transformer proposed by Liang et al. [33]; in SAC-B-CNN, we use the convolution with large kernel for EPI to extract features proposed by Xia et al. [12].

**Figure 13:**
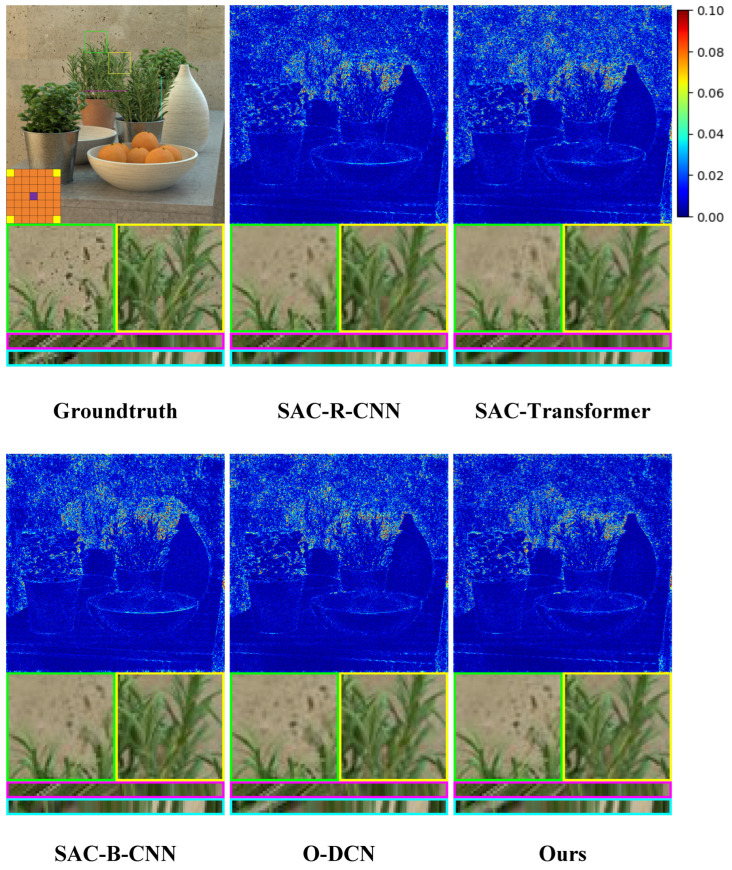
The qualitative results of the ablation study to verify the effectiveness of the MMOF-DCN and the MMOF design for DCN are shown. We select the HCInew_dishes [30] and show it with the central view of (4,4). The results show the error map, two local details, a H-EPI and a V-EPI.

As shown in Table 6, the PSNR values of these three variants decreased by 1.25 dB, 0.88 dB, and 0.65 dB, respectively, compared to our proposed method. In addition, as shown in Figure 13, the H-EPI and V-EPI reconstructed by the three variants are less clear than those reconstructed by our proposed method. Both quantitative and qualitative results demonstrate that the DCN-based approach is more effective in addressing the issue of varying and inconsistent pixel distances required for extracting SAC features.

#### 5.3.2. Effectiveness of Multi-Maximum-Offsets Fusion (MMOF) Design for DCN

To verify the effectiveness of the MMOF design for DCN, we introduced a variant named O-DCN. Specifically, O-DCN follows the original design by Wang [13], where a DCN is first applied, followed by a Linear layer to enhance extracted features. In O-DCN, we use the maximum offset value from the LF images in the synthetic dataset to ensure the exploration of SAC features at the greatest possible distance.

As shown in Table 6, the PSNR value of O-DCN is lower by 0.33 dB compared to our proposed method. This quantitative result suggests that the MMOF design effectively extracts SAC features not only at greater distances but also accurately at shorter and medium distances. In addition, as shown in Figure 13, the reconstruction of spots on the wall by O-DCN is not as clear as that of our proposed method, which proves that the MMOF design can reconstruct more details.

#### 5.3.3. The Number of DFE Blocks Used

The DFE is composed of *m* DFE Blocks, where in our proposed method, m=3. To validate the appropriateness of this strategy, we experimented with different values of *m* and analyzed the results in terms of both parameter and quality.

**Figure 14:**
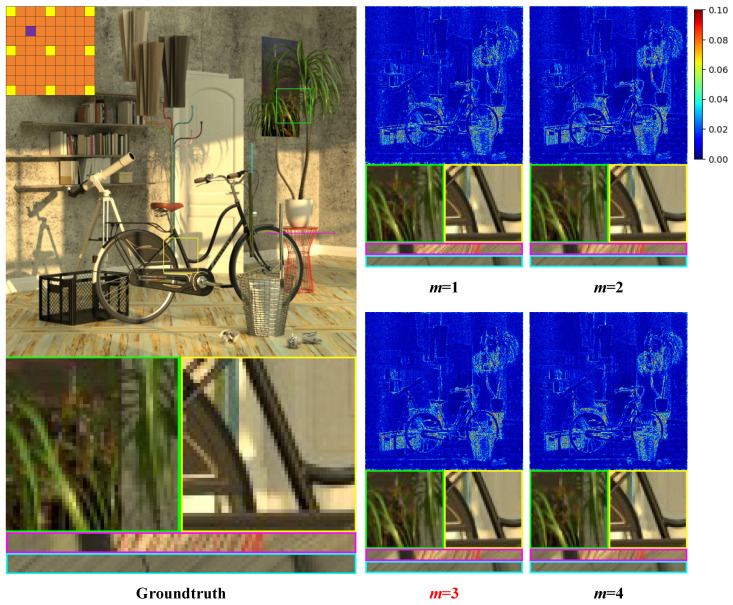
The qualitative results of the ablation study validate the most reasonable number of DFE blocks. We select the HCInew_bicycle [30] and show it with the central view of (4,4). The results show the error map, two local details, a H-EPI and a V-EPI. Here, *m* represents the number of DFE blocks, and we propose that m=3 be highlighted in red.

As shown in Table 7, when m=1 or m=2, the PSNR values decrease significantly compared to when m=3. For values of *m* greater than 3, the increase in PSNR is minimal, while the parameter rises substantially. In contrast, when m=3, the PSNR value reaches its optimal level, and the parameter count remains moderate. In addition, as shown in Figure 14, when m=1 or m=2, the errors in the error map is very obvious, and the shape of the circular projection in the local details is incomplete. When m=3 and m=4, the quality of the reconstructed image is significantly improved, and the quality becomes nearly identical. Therefore, we conclude that using m=3.

## 6. Conclusions and Future Work

In this paper, we address the limitations of existing LFASR methods, where inaccuracies in SAC feature extraction lead to insufficient reconstruction of disparity structures, resulting in blurred regions and artifacts. To overcome these challenges, we proposed MMOF-DCN. Our approach begins with an analysis of two key challenges in SAC feature extraction for LAR LF images: (1) the distance between pixels required for SAC feature extraction increases and the number of pixels is limited, and (2) the inconsistent distances necessary for extracting different SAC features. These challenges make it difficult to achieve accurate and comprehensive SAC feature extraction. To address the first issue, we employ DCN to adaptively adjust sampling point positions, thereby expanding the sampling range to handle larger pixel distances. For the second issue, we introduce a multi-maximal offset fusion mechanism within DCN to accommodate the varying pixel distances required for different SAC features. We conducted extensive experiments on both real-world and synthetic datasets. The results demonstrate that our proposed method achieves superior quantitative and qualitative performance compared to existing methods. Notably, in challenging tasks and synthetic LF scenes with large disparities, our proposed method achieves a PSNR improvement of 0.45 dB over existing methods. Ablation study further confirm the effectiveness of each module in our proposed method.

Despite our proposed method has shown the best reconstruction of HAR LF images, our work primarily focuses on accurate SAC feature extraction with limited exploration of spatial and angular features. We recognize that leveraging spatial and angular features more effectively can enhance detail and texture clarity in reconstructed LF images. In future work, we plan to investigate the properties of spatial and angular features in LAR LF images more comprehensively and design improved network architectures to optimize feature extraction.

## Figures and Tables

**Table 1:** For the 2×2→7×7 LFASR task, the results of the comparative experiment on real-world dataset. We use PSNR/SSIM to evaluate. The best result in red and the suboptimal result in blue.

Method	30Scenes	Occlusion	Reflective	Average
Kanlantari et al. [14]	41.40/0.982	37.25/0.972	38.09/0.953	38.91/0.969
Yeung et al. [7]	42.77/0.986	38.88/0.980	38.33/0.960	39.99/0.975
Jin et al. [15]	42.54/0.986	38.53/0.979	38.46/0.959	39.84/0.974
SAANet [9]	40.95/0.981	36.75/0.971	37.85/0.955	38.51/0.969
DistgASR [10]	43.67/0.995	39.46/0.991	39.11/0.978	40.74/0.988
Xia et al. [12]	44.35/0.996	40.03/0.992	39.73/0.982	41.37/0.990
Ours	44.41/0.997	40.07/0.992	39.85/0.983	41.44/0.991

**Table 2:** For the 2×2→7×7 LFASR task, the results of the comparative experiment on synthetic dataset. We use PSNR/SSIM to evaluate. The best result in red and the suboptimal result in blue.

Method	HCInew	HCIold	Average
Kanlantari et al. [14]	32.85/0.909	38.58/0.944	35.71/0.926
Yeung et al. [7]	32.30/0.900	39.69/0.941	35.99/0.920
Jin et al. [15]	34.60/0.937	40.84/0.960	37.72/0.948
SAANet [9]	31.39/0.882	39.03/0.926	35.21/0.904
DistgASR [10]	34.70/0.974	42.18/0.978	42.18/0.978
Xia et al. [12]	36.35/0.981	42.56/0.983	39.46/0.982
Ours	37.07/0.985	42.85/0.984	39.91/0.985

**Table 3:** For the 3×3→9×9 LFASR task, the results of the comparative experiment on real-world dataset. We use PSNR/SSIM to evaluate. The best result in red and the suboptimal result in blue.

Method	30Scenes	Occlusion	Reflective	Average
SAANet [9]	43.85/0.994	38.78/0.978	42.37/0.988	41.67/0.987
DistgASR [10]	44.53/0.996	43.20/0.995	43.12/0.991	43.45/0.994
Xia et al. [12]	44.96/0.997	43.35/0.995	43.20/0.991	43.84/0.994
Ours	45.04/0.998	43.39/0.995	43.34/0.991	43.92/0.994

**Table 4:** For the 3×3→9×9 LFASR task, the results of the comparative experiment on synthetic dataset. We use PSNR/SSIM to evaluate. The best result in red and the suboptimal result in blue.

Method	HCInew	HCIold	Average
SAANet [9]	33.68/0.925	39.57/0.946	36.63/0.936
DistgASR [10]	37.53/0.983	42.51/0.977	40.02/0.980
Xia et al. [12]	37.85/0.984	42.76/0.980	40.31/0.982
Ours	38.22/0.987	42.92/0.981	40.57/0.984

**Table 5:** The number of parameters and reconstruction quality of our method are compared with existing methods. We used 2×2→7×7 LFASR task and synthetic dataset for comparative experiments. The best result in red.

Method	Params.	Synthetic Average
Yeung et al. [7]	1.50 M	35.99/0.920
Jin et al. [15]	1.11 M	37.72/0.948
SAANet [9]	0.34 M	35.21/0.904
DistgASR [10]	2.68M	38.44/0.976
Xia et al. [12](C=32)	1.53 M	38.41/0.975
Xia et al. [12] (C=64)	5.98 M	39.46/0.982
Ours (C=32)	1.47 M	38.74/0.977
Ours (C=64)	5.79 M	39.91/0.985

**Table 6:** The results of the ablation study to verify the effectiveness of the MMOF-DCN and the MMOF design for DCN. We use PSNR/SSIM to evaluate. The best result in red and the suboptimal result in blue.

Variant	HCInew	HCIold	Average
SAC-R-CNN	35.30/0.977	42.01/0.977	38.66/0.977
SAC-Transformer	35.87/0.979	42.19/0.980	39.03/0.980
SAC-B-CNN	36.20/0.980	42.32/0.982	39.26/0.981
O-DCN	36.66/0.982	42.50/0.983	39.58/0.983
Ours	37.07/0.985	42.85/0.984	39.91/0.985

**Table 7:** Parameters and qualities of different number of DFE blocks. We use PSNR/SSIM to evaluate. Our proposed m=3 is shown in bold.

Variant	Params	HCInew	HCIold	Average
m = 1	2.97 M	35.49/0.978	42.09/0.978	38.79/0.978
m = 2	4.31 M	36.24/0.981	42.31/0.982	39.28/0.982
**m = 3**	**5.79 M**	**37.07**/**0.985**	**42.85**/**0.984**	**39.91**/**0.985**
m = 4	7.10 M	37.16/0.985	42.91/0.984	40.03/0.985

## Data Availability

The original contributions presented in this study are included in the article. Further inquiries can be directed to the corresponding author.

## Abbreviations

The following abbreviations are used in this manuscript:

SSIM	Structural Similarity
PSNR	Peak Signal Noise Ratio
